# Byways of Medical Relief
*Previous articles appeared on Oct. 7 and 21, Nov. 18, Dec. 2, Jan. 6, Feb. 10 and 17, March 2 and 16.


**Published:** 1912-03-30

**Authors:** 


### BYWAYS OF MEDICAL RELIEF.*

(Contributions are invited to this column.)

### X.—Some Flaws in Little Homes.

The management of the homes we have briefly passed in review varies from the simplicity of a benevolent despotism to the complicated structure adopted by larger institutions, involving president, council, committee, and honorary officials. In starting little homes it is well to bear in mind that they may grow out of all likeness to their originals. Hence the foundation, however simple, cannot be too well laid, and sound principles should be adopted of finance and superintendence.

### Slipshod Finance.

It is astonishing how many public bodies are found, even in these days of efficient management, ignoring the elementary duty of issuing to subscribers a yearly report and statement of accounts. There is no home so small that it should break this great standing rule for all who attempt to benefit others by collective action. It is an offence against the public code of just business methods to omit to issue a plain statement showing the subscribers what has been done with their money. To prepare this statement, which need not be printed, it is essential to keep accounts. Nothing better for the purpose has ever been compiled than "The Uniform System of Accounts, Series 2, for Cottage Hospitals and Smaller Institutions." But the very sight of a properly ruled account-book on the double entry system is distressing to the mind of the amateur, and it may suffice for those who manage very small establishments to keep the following rules: First, to give a numbered receipt, retaining counterfoil, for every subscription, however small, including the grants made by the person who is actually conducting the home, and also to keep a list of all payments made by inmates. Thus at any time the income of the home can be readily ascertained. Second, to make all payments by cheque, including the sums afterwards to be accounted for under the head of "Petty Cash." Thus rent, doctor's bill, drugs, etc., can be paid direct by cheque. And every week, or every month, a cheque should be drawn for petty cash to cover the domestic expenses, wages, etc., and this amount should be strictly accounted for by tradesmen's books or vouchers. If this principle be carried out the cheque book shows the exact expenditure of the home, and a friendly auditor will find no difficulty in verifying the receipts and payments at the end of the year. An effort ought to be made to regulate the domestic expenses, so that one week can be compared with another; and when the numbers fluctuate, as in convalescent homes, the matron should keep a note of the number dining every day, so that at the end of the week an estimate may be prepared showing the average cost of each inmate of the home. This system is one which a thrifty matron delights in, as it shows up her good housekeeping and produces a welcome feeling of security that all is going well. It is all too common for money to be spent lavishly on the little homes without anyone being very clear as to what is actually obtained for it; and it is also fairly common for the matron to be pinched for the most ordinary necessities because the managers have never taken the trouble to make out a definite budget and do not understand what is a fair average expenditure.

### Slipshod Superintendence.

The greatest of all difficulties in little homes is to get the right person to act as matron or superintendent. The success of the whole enterprise depends upon this, and the mistakes which are often made in management start from faulty methods of choosing the matron. The great blunder is to take it for granted that a friend of one of the subscribers is more likely to be the right person than a total stranger. A kind of word painting is very common in describing the qualifications of acquaintances to whom one desires to do a good turn, and this is responsible for foisting many an excellent woman into a post for which she is not in the least fitted. Committees of management are oddly given to shirking the onus of selecting candidates from answers to advertisements, although the form of the testimonials supplied is ample guarantee, if supplemented by personal interviews, against imposture. When once the matron is chosen the managers are liable to two very different mistakes in directing her. Either she is treated with a blind confidence which rejects inspection as a sort of insult and can see no possible defect, or she may be exposed to very irritating surveillance from several members of a committee. Either course will prejudice the success of the home, and it depends on the character of the matron herself which will prove the more harmful. One person only should be entitled to comment on the matron's management. Free inspection of premises and domestic arrangements should be a matter of course, and confidence should be encouraged between the honorary official and the matron, so that difficulties can be peacefully discussed and the burden of responsibility be lessened. When things go wrong, when complaints of patients continue, and a condition of discontent is noted, there should be a change of matron. Managers are very apt to drift on long after it has become perfectly clear that no one is satisfied, and that the home is suffering. It is wiser by far to face the situation, open the matter plainly to the matron, and recommend her to resign, always taking heed to give time and opportunity for seeking another post. Something better than bare legality is due from the manager of a charity to those who assist, even unsuccessfully, in carrying out their aims. It has been our lot to witness more than one charity planned with admirable wisdom falling short conspicuously of its purpose for want of courage in altering the management.

* Previous articles appeared on Oct. 7 and 21, Nov. 18, Dec. 2, Jan. 6, Feb. 10 and 17, March 2 and 16.

